# Do Pigs Have Adequate Space in Animal Transportation Vehicles?—Planimetric Measurement of the Floor Area Covered by Finishing Pigs in Various Body Positions

**DOI:** 10.3389/fvets.2018.00330

**Published:** 2019-01-10

**Authors:** Heidi Arndt, Nina Volkmann, Birgit Spindler, Jörg Hartung, Nicole Kemper

**Affiliations:** ^1^Clinic for Cattle, University of Veterinary Medicine Hannover, Foundation, Hannover, Germany; ^2^Institute for Animal Hygiene, Animal Welfare and Farm Animal Behaviour (ITTN), University of Veterinary Medicine Hannover, Foundation, Hannover, Germany

**Keywords:** body dimensions, image analysis, loading density, physical space, stocking densities

## Abstract

In this study, the floor area covered by individual finishing pigs in various body positions was measured using a contrast-based planimetric method for computer-assisted analysis of two-dimensional images. Two hundred and thirty-two finishing pigs were weighed during the last fifth of the fattening period and measured in different body positions using contrast-based planimetry. Thirteen body positions were defined based on characteristic directions of the head, legs and body. The lowest average covered floor area was found for body position A (pig standing up straight, nose touching the ground) with 0.288 ± 0.026 m^2^. The highest average covered floor area for a standing pig amounted to 0.335 ± 0.030 m^2^ in body posture ES (pig standing curved sideways, head raised above the dorsal line) and, for a lying pig, 0.486 ± 0.040 m^2^ (posture LL, pig lying in fully lateral recumbent position). The covered floor surface significantly depended on the weight of the animal and the body posture. Allometric estimations previously described for calculating the floor area physically covered by a pig's body are not consistently precise in depicting the actual areas covered. The minimal floor area offered in animal transportation vehicles, according to European legislation, is insufficient in the case of all pigs lying in the fully recumbent position simultaneously, without the pigs being forced to partially overlap one another. Therefore, both allometric formulas and legislation should be modified on the basis of these results and further studies with pigs of modern genetic origin should be conducted.

## Introduction

In the European Union about 255 million pigs are slaughtered each year (1). Most of these are fattening pigs transported to the slaughterhouse by trucks. During these journeys, fattening pigs are exposed to numerous and variable stressors, such as variations in ambient temperature, vehicle movements, handling by humans, social pressure by mixing unfamiliar pigs or inappropriate space available (2–4). It is generally accepted that the loading density on the transport vehicle has a strong influence on the welfare of the pigs and that the provision of appropriate space is a key factor (5). To provide recommendations for statutory space requirements, the floor area covered by a pig's body (referring to the static space) requires a precise definition. This can either be calculated with mathematical formulas by means of allometric principles (6–9), or determined using data collected directly on the animal's body or measured indirectly using image analysis (10, 11). Allometric formulas were drawn up based on measurements of length, width and height of whole animals' bodies, or their body parts. In pigs, these measurements were carried out by Petherick and Baxter (12) in the 1980s and the derived allometric formulas were designated by the authors as a “good starting point” (13). However, on-going technological progress has enabled more precise methods, such as computer-based planimetric image analysis, which considers the animals' accurate actual body outline, as shown in poultry (14, 15). Methods for area calculation from two-dimensional images of pig body dimensions were also tested for various fields of application (10, 16–18). However, data from various genetics or weight classes for the verification of allometric estimations or recommendations for loading densities are lacking.

Accordingly, current European legislation is based on outdated data. In Council Regulation (EC) No 1/2005 (19) for pigs of around 100 kg, a maximum of 235 kg live weight per m^2^ floor area on a road transport vehicle is given. This is intended to ensure that all pigs are able to stand and to lie in their natural body position. The value of 235 kg/m^2^ has been adopted from the Council Directive 95/29/EC (20) without modifications, because the member states were not able to agree on a revision of stocking densities until the adoption of the current regulation. Clarification of this issue in a separate proposal, written within 4 years of coming into effect, was decided but is missing until today (21). Originally, the value of 235 kg/m^2^ was based on recommendations by Lambooy et al. (22), who rated this minimum loading density for finishing pigs as a compromise of “animal welfare, meat quality aspects and economy of transport.” Thus, the base for loading densities recommended by European legislation dates back a few decades. Since then, the genetics in fattening pigs, not only in Germany, have changed, resulting in an increase of slaughter weights from around 83 kg in 1984 to 95 kg nowadays (23), and live weights increased from around 100 kg to around 110 kg or more. This should influence the spatial needs considerably, although the regulation also states that depending on the breed, size and physical condition of the pigs, the weather and the journey time, an undefined increase of up to 20% of the minimum floor area may be required. There lacks, however, a more precise definition.

The aim of this study was to determine the actual floor taken up by finishing pigs of a modern pig hybrid type in different body positions. An existing computer-assisted planimetric image analysis method was modified for application on fattening pigs. By considering measured values according to the live weight, and comparison with values calculated by allometric formulas, the accuracy and practicability of existing allometric formulas estimating the floor area physically occupied by an individual pig's body were assessed. Based on the static space measurements, a further objective was to examine whether sufficient space on road transport vehicles is provided to fattening pigs in the actual European regulation if the minimum requirements are met.

## Materials and Methods

### Animals

A total of 232 modern finishing pig hybrids (108 ♂ neutered and 124 ♀) that are genetically prevalent in Europe (Danish Breed Sows x Pietrain boar) were weighed within the last fifth of the fattening period, representing the weights expected on animal transport vehicles, and measured by contrast-based planimetry. The measurements were carried out on one farm with two compartments, each with 200 fattening places in total in 13 trial days from June until November 2013. All animals originated from the farm's own piglet production. All animals in this study were kept in accordance with European Union guidelines (24). The protocol was approved by the University's Animal Protection commissioner.

### Experimental Setup for the Assessment of Area Values

For the determination of the static space, the KobaPlan method (11), until now mainly used for surveying poultry, was used for computer-assisted analysis of two-dimensional images (11, 14, 15). The method setup had to be adapted for application on fattening pigs. A planimetry box (245.00 × 125.00 × 120.00 cm) was constructed. To prevent slipping, the base plate consisted of an aluminum checker plate (125.00 × 245.00 cm, thickness 4.0 mm). The side panels were built of solid wooden material (thickness: 9.0 mm) to resist the occasionally rough exploratory behavior of pigs. To enhance the contrast between background and animal, the base and side plates were painted with fluorescent color, the surrounding area was darkened and the box was lighted with ultraviolet light [Omnilux UV- energy-efficient lamp: 85 W (2x), 25 W (2x); Omnilux, Waldbüttelbrunn, Germany and Eurolite UV- neon tube; Eurolite, Waldbüttelbrunn, Germany]. A digital SLR camera (Canon EOS 600D; Canon Deutschland GmbH, Krefeld, Germany) with a standard camera lens (18-55 IS II; Canon Deutschland GmbH, Krefeld, Germany) was fixed centrally above the box and focused on the bottom plate. The distance was 245.00 cm between the bottom surface and the lens. The camera was connected to a notebook via a serial USB interface. Using control software (EOS Utility, Canon Deutschland GmbH, Krefeld, Germany) a live image was transferred, and the camera was triggered via the notebook keyboard manually.

First the current live weight of each individual pig was documented by weighing with a livestock scale (Box livestock scales; Baumann Waagen- und Maschinenbau GmbH, Thiersheim, Germany). Then, the animal was led into the planimetry box where various two-dimensional plan top view photographs were taken under constant shooting conditions (focal length 18.0 mm, consistent focus) (Supplementary Figure 1). An attempt was made to photograph each animal in 10 standing and three lying body positions, chosen with the intent to reflect the natural movement of pigs. Later, one single characteristic image of each pig was chosen for each available position. The 13 positions, explained by exemplary images, the numbers of pictures for each position, information on gender and weight structures of the total data set are listed in Table 1.

**Table 1:** Description of evaluated pigs' body positions, number of analyzed individual pictures (*n*; n_total_ = 1,583 from 232 pigs) for each position, gender (tot = total sample; tot_fem = females of total sample; tot_mn = neutered males of total sample), and live weights (Mean, SD = standard deviation, Min = Minimum, Max = Maximum); ^a,b^Columns with different superscripts differ significantly (*p* < 0.05).

**Position**			**Gender**	***n***	**Live weight (kg)**			
					**Mean**	**SD**	**Min**	**Max**
Total			tot	1,583	109.01	11.45	75.00	133.00
			tot_fem					
			tot_mn					
A	Standing up straight, nose touching the ground	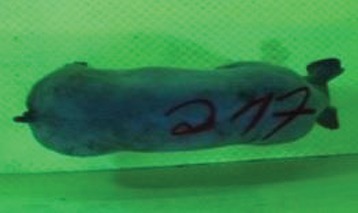	tot	185	109.09^ab^	11.23	76.00	133.00
			tot_fem	99	109.14	11.22	76.50	133.00
			tot_mn	86	109.03	11.31	76.00	132.50
AS	Standing curved side-ways, nose touching the ground	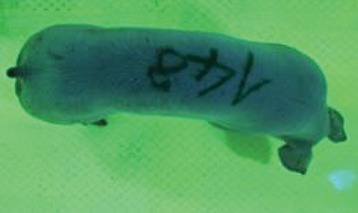	tot	138	110.58^ab^	10.39	76.50	133.00
			tot_fem	79	109.75	11.15	76.50	133.00
			tot_mn	59	111.70	9.25	90.00	132.50
B	Standing up straight, head lowered to the ground	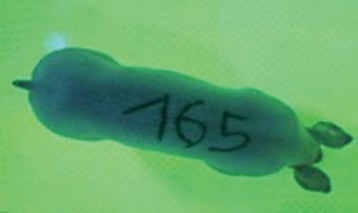	tot	154	108.13^ab^	11.57	76.00	133.00
			tot_fem	76	108.47	11.95	76.50	133.00
			tot_mn	78	107.79	11.26	76.00	127.00
BS	Standing curved sideways, head lowered to the ground	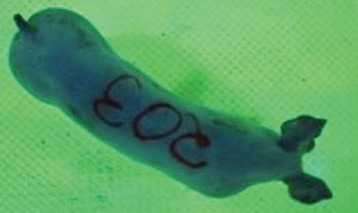	tot	108	110.98^ab^	10.14	76.50	133.00
			tot_fem	75	109.66	10.52	76.50	133.00
			tot_mn	33	113.99	8.62	90.00	130.00
C	Standing up straight, head raised below the dorsal line	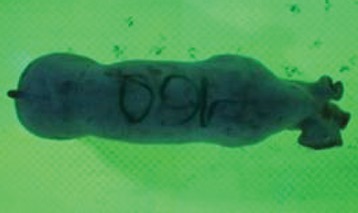	tot	181	109.07^ab^	11.92	75.00	133.00
			tot_fem	95	109.30	12.21	75.00	133.00
			tot_mn	86	108.82	11.67	76.00	132.50
CS	Standing curved side-ways, head raised below the dorsal line	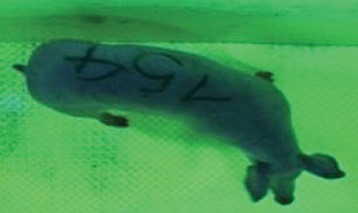	tot	151	111.22^a^	10.59	76.50	133.00
			tot_fem	86	109.61	11.60	76.50	133.00
			tot_mn	65	113.35	8.72	90.00	130.00
D	Standing up straight, head raised at the level of dorsal line	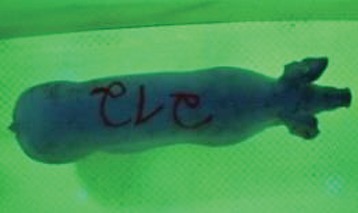	tot	201	108.08^ab^	11.38	75.00	131.00
			tot_fem	107	108.23	11.56	75.00	131.00
			tot_mn	94	107.90	11.23	76.00	127.00
DS	Standing curved side-ways, head raised at the level of dorsal line	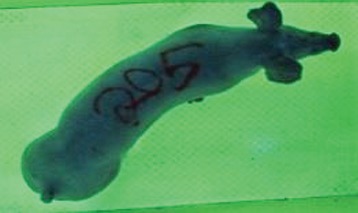	tot	156	108.62^ab^	11.45	75.00	131.00
			tot_fem	86	108.04	11.30	75.00	131.00
			tot_mn	70	109.34	11.67	76.00	125.50
E	Standing up straight, head raised above the dorsal line	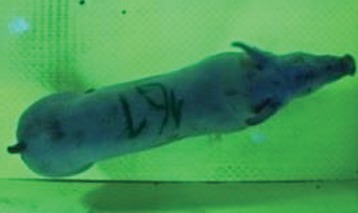	tot	112	106.26^b^	12.52	75.00	131.00
			tot_fem	51	105.02	12.31	75.00	131.00
			tot_mn	61	107.30	12.71	76.00	127.00
ES	Standing curved side-ways, head raised above the dorsal line	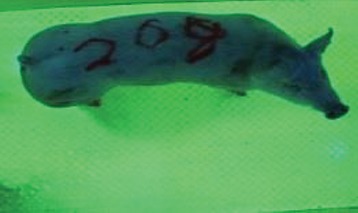	tot	68	110.48^ab^	11.82	75.00	133.00
			tot_fem	37	107.82	13.90	75.00	133.00
			tot_mn	31	113.65	7.83	100.50	125.50
LBC	Lying in sternal (belly chest) recumbency	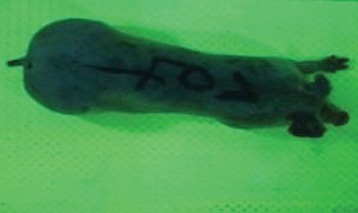	tot	50	108.21^ab^	11.62	86.00	132.50
			tot_fem	26	109.67	9.92	90.00	131.00
			tot_mn	24	106.63	13.26	86.00	132.50
LSL	Lying in semilateral (lateral chest) recumbency	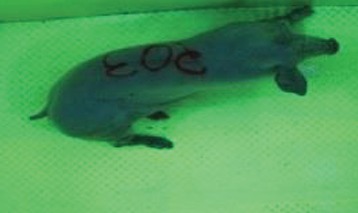	tot	49	106.89^ab^	10.36	86.00	131.00
			tot_fem	25	109.52	9.95	90.00	131.00
			tot_mn	24	104.15	10.26	86.00	124.00
LL	Lying in lateral (full) recumbency	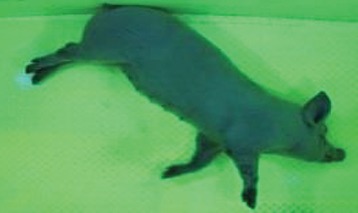	tot	30	107.50^ab^	10.54	86.00	132.50
			tot_fem	13	111.04	8.90	101.50	131.00
			tot_mn	17	104.79	11.14	86.00	132.50

### Image Analysis

KobaPlan software (11) was used in version “KobaPlan v.01.teta” (© Briese 2007–2013, eduToolbox@Bri-C GmbH, Sarstedt, Germany). On the basis of the contrast between the animal and its environment, the area occupied by the animal's body was calculated automatically by the software. For this, a reference surface was formed by a planar rectangular wooden board with an extent similar to a fattening pig body in supervision (0.420 m^2^). This reference surface was used to calculate the relation between known area and number of pixels as base for the assessment of the area occupied by the animal's body. To analyze the images of standing pigs, the reference was mounted at a height of 69.00 cm, which was the mean height by measuring pigs in standing position using a stick measuring device and a folding rule (*n* = 348, height 68.54 ± 10.69 cm). For lying pigs, a mean height of 29.00 cm (*n* = 171, height 29.44 ± 5.90 cm) was measured with the same procedure. The reference surface was photographed under the same conditions as the animals, and information about size and the dimensions on the picture were imported to the software. Additionally, the program created a copy of the original image, which showed the calculated area colored blue for the visual verification of the recognition accuracy. About one third of the images were recognized without further processing, and two thirds had to be adapted using photo editing software (Adobe Photoshop CS6, Adobe Systems GmbH, Munich, Germany). In many cases, a subsequent increase in contrast was sufficient to ensure correct recognition of images. Shadows, feces or other contamination of the planimetry box had to be partly retouched to enable detection.

### Statistical Analyses

The measured data were described with basic descriptive statistical analysis parameters (averages, minimum and maximum values, standard deviations) calculated by the Excel program (Microsoft EXCEL 2014, Microsoft Corporation, Redmond, USA). Further statistical analyses were performed using SAS 9.4 (SAS Institute Inc., Cary, USA). For examining normal distribution, the procedure PROC UNIVARIATE was used. The differences of weights between the groups of position were tested with PROC GLM and the Tukey method was applied for the least square means. Comparisons of means were carried out using the PROC TTEST. Furthermore, the Spearman's correlation between mean weights and the covered floor area within the groups of body position were calculated using the SAS procedure PROC CORR. Results were considered statistically significant if the related *p*-values were <0.05.

### Comparison With Results From Allometric Formulas for the Calculation of the Floor Space Physically Covered

Values calculated on the basis of recommended allometric formulas taken from the review of Petherick and Phillips (25) were compared with the corresponding measured values. For the calculation of the covered floor area of single pigs while “standing” and while “lying on sternum/belly with legs folded beneath the body” the same allometric formula was recommended: a = 0.019 W^0.66^ m^2^ (a = covered area, W = live weight) (25). As W, the mean weight assessed for the different body position groups was used. The floor areas needed for pigs of both postures were calculated by this formula and were compared with the planimetric values measured for pigs in different standing body positions and with those measured for the LBC position [“lying in sternal (belly chest) recumbency”]. The values calculated for “semirecumbent lying” [formula according to Petherick and Phillips (25): a = 0.025 W^0.66^ m^2^] were compared to those measured by planimetry for position LCL [“lying in semilateral (lateral chest) recumbency”], and for “fully recumbent lying” (formula: a = 0.047 W^0.66^ m^2^) with position LL [“lying in lateral (fully) recumbency”].

### Comparison With Recommended Legal Space Requirements on Transport Vehicles in Europe

The floor area values measured were set into relation to legal space requirements of Council Regulation (EC) No 1/2005 (19). Therefore, the average covered areas measured for each position group were compared with the minimum legal recommendations of 235 kg/m^2^, calculated for the average animal weights within the 13 body position groups.

## Results

### Measured Area Values

In total, 1,583 (n) pictures of 232 pigs were evaluated. The weight range was between 75.00 and 133.00 kg (mean ± standard deviation: 109.01 ± 11.45 kg; Table 1). Given that not all pigs could be assessed in all positions, we compared average body weights of position groups to make sure that there were no or only small differences between the groups, and therefore comparability exists. Only the mean weights for body position group E (106.26 ± 11.45 kg) and position group CS (111.22 ± 10.59 kg) varied significantly, while for all other position groups, no significant variations were detected (Table 1). Thus, the different area values between standing and lying animals can be related to the position and not to any weight differences.

**Figure 1:**
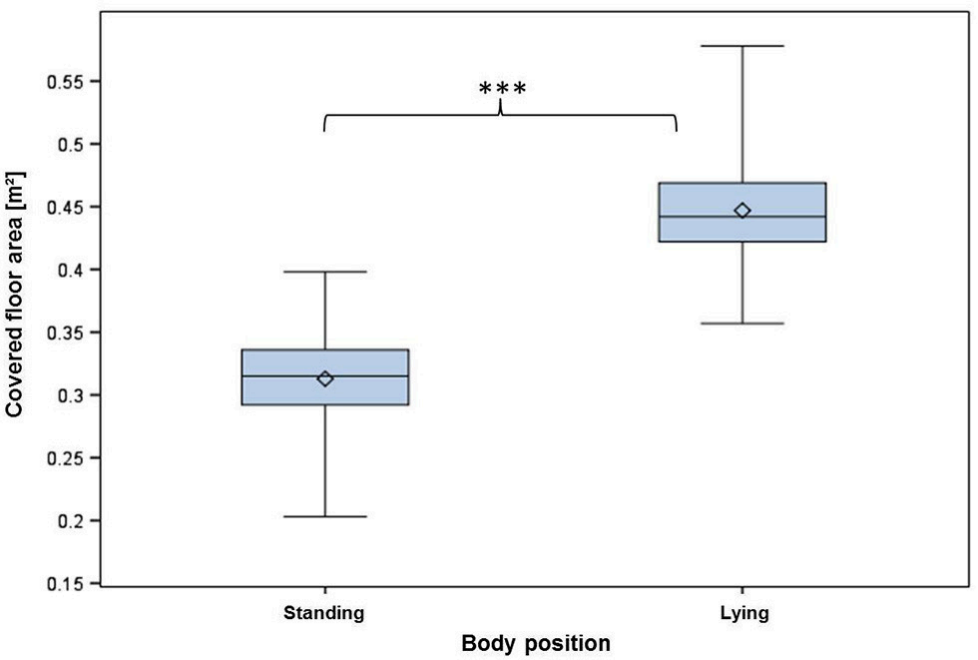
Covered floor area of pigs in standing and lying body positions; standing positions A-ES, lying positions LBC -LL (see Tables 1, 2), ^***^p < 0.001.

**Figure 2:**
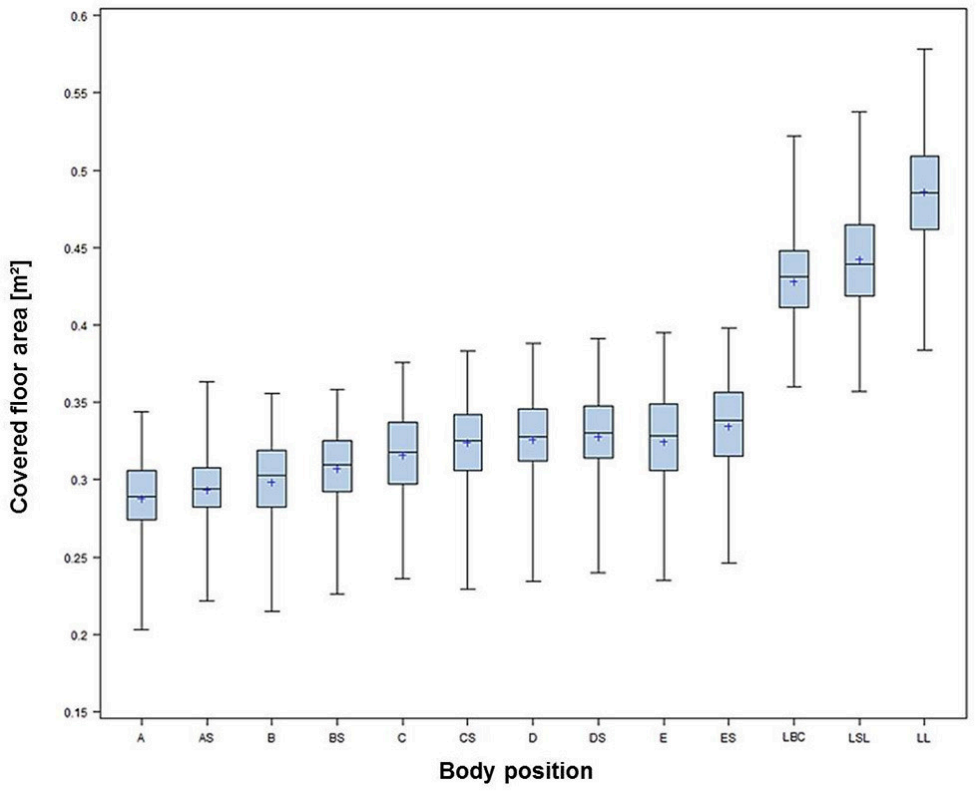
Covered floor area of pigs in body positions AL-LL (see Tables 1, 2).

The covered floor area depended on the animal's body position, as shown in Table 2. In general, lying positions required significantly more space than standing positions (Figure 1). In more detail, the assessed values for the different body position groups are reflected in Figure 2. In standing positions, the mean covered area values varied between 0.288 ± 0.026 m^2^ for position A (“Standing up straight, nose touching the ground”) and 0.335 ± 0.030 m^2^ for body position ES (“Standing curved sideways, head raised above the dorsal line”). In lying positions, the occupied space ranged between 0.428 ± 0.032 m^2^ for pigs in body position LBC [“Lying in sternal [belly chest] recumbency”] and 0.486 ± 0.040 m^2^ in body position L [“Lying in lateral (full) recumbency”]. The absolute minimum value of 0.203 m^2^ floor area physically covered by an individual pig was measured for a pig of 83.50 kg while standing in body position A. The absolute maximum value of 0.578 m^2^ was found for a pig of 131.00 kg lying fully recumbently in body posture LL (Table 2).

**Table 2:** Floor area (m^2^) physically covered by a fattening pig in various body positions (n_total_ = 1,583 pictures from 232 pigs; Mean, SD = standard deviation, Min = Minimum, Max = Maximum).

**Position**			**Covered area (m**^****2****^**)**			
			**Mean**	**SD**	**Min**	**Max**
A	Standing up straight, nose touching the ground	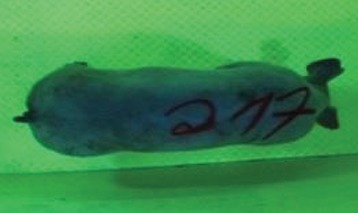	0.288	0.026	0.203	0.344
AS	Standing curved sideways, nose touching the ground	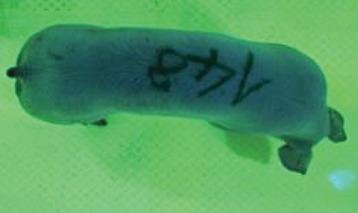	0.294	0.023	0.222	0.363
B	Standing up straight, head lowered to the ground	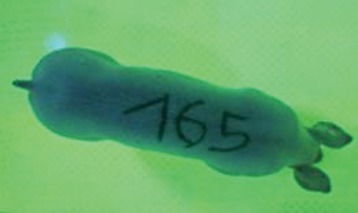	0.298	0.029	0.215	0.356
BS	Standing curved sideways, head lowered to the ground	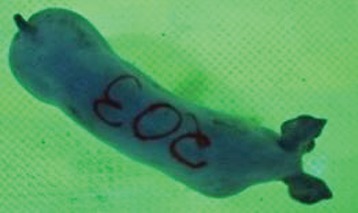	0.307	0.026	0.226	0.358
C	Standing up straight, head raised below the dorsal line	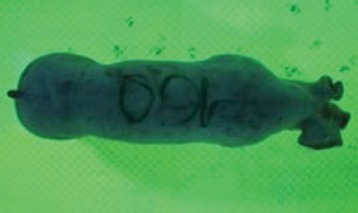	0.316	0.030	0.236	0.376
CS	Standing curved side-ways, head raised below the dorsal line	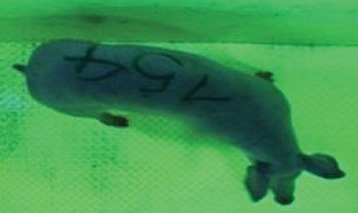	0.324	0.026	0.229	0.383
D	Standing up straight, head raised at the level of dorsal line	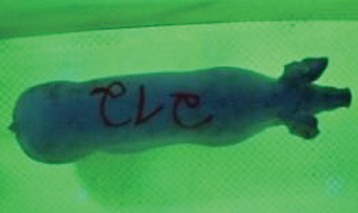	0.326	0.030	0.234	0.388
DS	Standing curved side-ways, head raised at the level of dorsal line	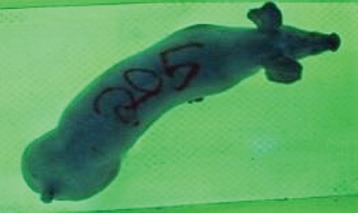	0.328	0.030	0.240	0.391
E	Standing up straight, head raised above the dorsal line	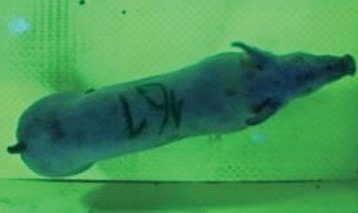	0.325	0.033	0.235	0.395
ES	Standing curved side-ways, head raised above the dorsal line	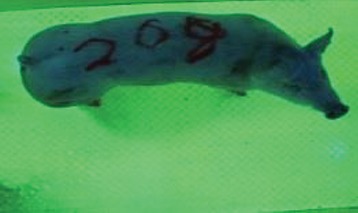	0.335	0.030	0.246	0.398
LBC	Lying in sternal (belly chest) recumbency	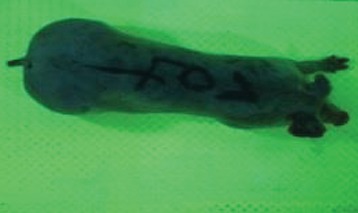	0.428	0.032	0.360	0.522
LSL	Lying in semilateral (lateral chest) recumbency	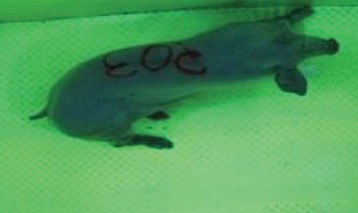	0.442	0.039	0.357	0.538
LL	Lying in lateral (fully) recumbency	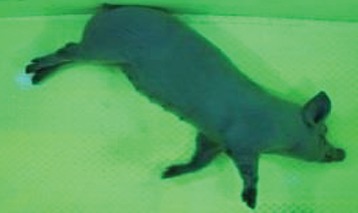	0.486	0.040	0.384	0.578

In all position groups, the covered floor surface depended significantly on the weight of the animal (*p* < 0.0001). Though areas corresponding to lying positions appear to have smaller correlation coefficients compared to standing positions, this could be related to the shape of the pigs and the mass of the hind quarters, but a final explanation cannot be given. Correlations between the covered floor area and live weight are represented in Table 3.

**Table 3:** Spearman's correlations between mean weights and covered floor area within the groups of body positions A-LL (see Tables 1,2); all results: *p* < 0.05.

	**A**	**AS**	**B**	**BS**	**C**	**CS**	**D**	**DS**	**E**	**ES**	**LBC**	**LSL**	**LL**
R	0.852	0.809	0.855	0.779	0.878	0.861	0.889	0.856	0.923	0.88	0.692	0.647	0.511

### Comparison With Results From Allometric Formulas for the Calculation of the Floor Space Physically Covered

**Figure 3:**
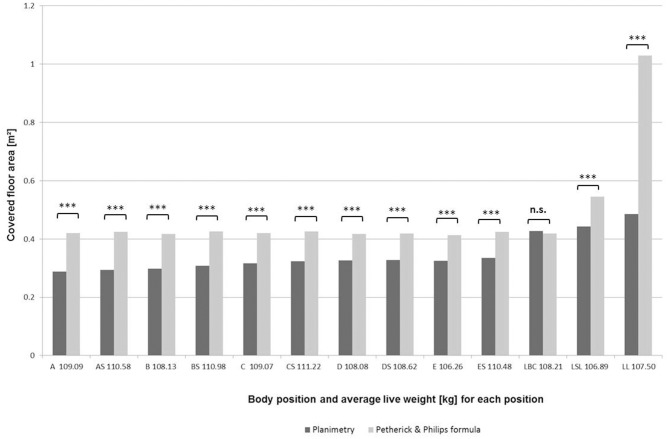
Measured values (m^2^) of floor area physically covered in various body positions (A-LL; see Tables 1, 2) in comparison with values calculated with allometric formulas by Petherick and Philips (2009) according to the body posture (a = 0.019 W^0.66^ m^2^ (a = covered area, W = live weight (kg) for “standing” and “lying on sternum/belly with legs folded beneath the body” according to position A-LBC; a = 0.025 W^0.66^ m^2^ for “semirecumbent lying” according to LCL; a = 0.047 W^0.66^ m^2^for “fully recumbent lying” according to LL) and the mean live weight of each body position group, ^***^p < 0.001.

The calculation of the covered floor space by allometric formulas, based on the respective mean live weights in the different body position groups resulted in areas between 0.41m^2^ (Position E) and 1.03 m^2^ [Position LL, “lying in lateral (full) recumbency”]. The comparison of the calculated covered areas according to Petherick and Phillips (25) for both standing and lying positions revealed that the values calculated were—with the exception of one position (LBC)—significantly above the average values assessed by planimetric measurements (Figure 3). For animals “lying on sternum/belly with legs beneath the body,” the calculated area was less than the mean area measured for position LBC with a deviation of 0.010 m^2^. For the ten standing postures (A-ES), the average deviation between calculated and measured areas was 0.107 m^2^ (± 0.017, minimum: 0.089 m^2^ for body position ES, maximum: 0.132 m^2^ for body position A). For animals lying semirecumbently (according to body position LCL), the calculated area was 0.103 m^2^ above the mean area actually measured. The highest deviation was found for animals in body position LL, with a 0.544 m^2^ difference between the calculated and measured space.

### Comparison With Recommended Legal Space Requirements on Transport Vehicles in Europe

The calculation of legal space requirements based on Council Regulation (EC) No 1/2005 (19)—without the 20% addition—showed minimum space requirements for the respective mean live weights in the different body position groups between 0.452 m^2^ (Position E) and 0.473 m^2^ (Position CS). Concerning these legal minimum requirements, no differentiation between different body positions is given.

**Figure 4:**
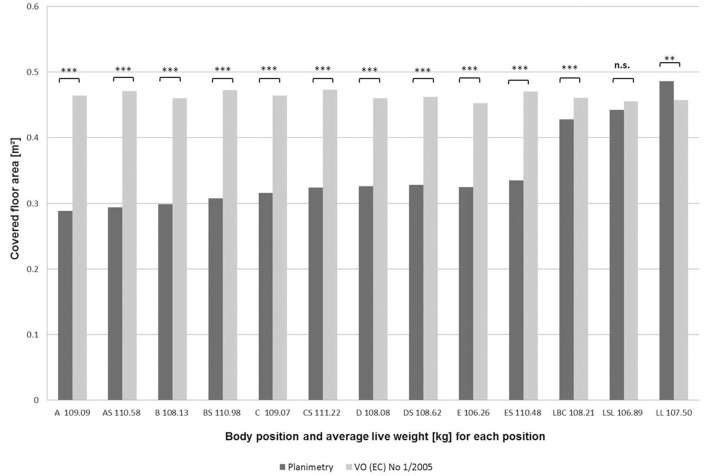
Measured values (m^2^) of floor area physically covered in various body positions (A-LL; see Tables 1, 2) in comparison with values calculated according to the space requirements from Council Regulation (EC) No 1/2005 (2004) (for pigs of around 100 kg” a maximum of 235 kg live weight per m^2^, adapted to the mean live weight of each body position group), ^**^p < 0.01, ^***^p < 0.001.

Comparing the averages of the measured floor areas by planimetry with the minimum space requirements, the measured covered areas for the static space were below the legally required minimums for 12 of the 13 positions, with the exception of body position LL, exceeding the legal recommendations by 0.028 m^2^. The other deviations varied between 0.012 m^2^ for body posture E and 0.177 m^2^ for body posture AS (Figure 4). Except for LBC, those deviations between measured and legally required space were significant. Deviations correspond to the floor space that remains available to an individual pig to carry out further movements, respectively, to sustain their individual distance. For instance, for a pig “lying in sternal (belly chest) recumbency” (LSL) a free area of 0.033 m^2^ remained, while in a fully recumbent body posture (LL), the floor space actually physically needed was above the minimum legal recommendation (Figure 4).

### Discussion

Commercial transport is a complex stressor with potential negative consequences for animal welfare (26–28). The impact on animal welfare is related to different factors with various and often not clearly defined impacts, and space is one of the most important influencing factors. The precise determination of space requirements is essential. The minimum space requirement of an animal is given by its physical dimensions, defined as static space. One aim of this study was to assess the static space of finishing pigs in different body positions by planimetry. By applying this method, our study supports the approach of calculating floor area needs based on the animal's weight and body position, which is supported by previous scientific studies (25) and is a legal requirement (19).

The limitation of such planimetric measurements is that additional demands, defined by dynamic and social space, are disregarded. While static space takes the body dimensions of the pig into account, dynamic and social space include the additional space needed for (non-) locomotor body movements and social interactions, respectively (7, 18). Moreover, the applied planimetric measurement is on a single animal basis and does not take social group aspects such as huddling into account.

However, as a minimum requirement, the static space is of essential importance. The planimetric method used in this study is an established and well-tried method for different animal species, including chickens, rabbits and piglets (15, 29–32), but needed to be modified for application on fattening pigs. This modification worked well and delivered good results, even though modern technologies, for instance three-dimensional cameras, offer further, less labor-intensive options (33, 34). With the modified planimetric method, the assessment of 13 different body positions was realized. The consideration of different standing and lying positions is of special importance for the recommendations of space requirements on transportation vehicles, especially for long-distance transports. Pigs on transport vehicles should be able to rest simultaneously (35). Therefore, not only the space for standing, but also for lying positions had to be considered. In this study, for the first time, data of the actual floor area physically needed by finishing pigs of current genetics for different positions are represented. Comparable data on static space covered by a pig's body from older studies are rare, for instance McGlone and Pond (36) stated that a lying pig in the lateral position of around 100 kg covers 0.56 m^2^, which is about 0.08 m^2^ more than in our study for the respective position.

The larger body size of genetically modern pigs is also reflected in increased mean weights. The assessed variation between 75.00 and 133.00 kg allowed an analysis for correlations between body weight and allocated space. It was shown that the floor area covered by an individual fattening pig body depends mainly on its weight, and on the pig's body position, as other studies had already reported (6, 10). This confirmed correlation between weight and space reveals the importance of adapting recommendations for space requirements, such as Council Regulation (EC) No 1/2005 (19), based on conditions over 30 years ago, to the realities of transporting modern finishing pigs. Concerning the results for the allocated space in different body positions, lying pigs, especially in lateral recumbency, need significantly more space than standing ones. This fact is of critical importance with regards to determining the required space in transportation vehicles, as all pigs should be able to lie down (35). With the values determined in our study, this can be assumed not only for lying fully recumbent, but also for lying in half recumbency there is little space. For higher weights (130–135 kg), exceeding the limit values for semi-recumbent lying is conceivable. Depending on the ambient temperature and group composition, animals may be either dense or loose together. However, it can be assumed that the problem of “too much” space is more applicable to standing animals, although the literature does not provide adequate explanations. A central problem with the determination of space requirements in transport vehicles, especially on long-distance-journeys, is the animals' behavior and, following, the problem of too much or too less space. It can be assumed that pigs take a standing position at the beginning of the journey and lie down at a later time to rest. This is the reason why sometimes for short journeys less space requirements are suggested. To our knowledge, no studies are available describing the behavior of pigs on transport vehicles sufficiently to derive representative durations of “standing periods” and “lying periods,” especially because those are influenced by many other factors. For that, further comprehensive studies are needed.

As the measurement of allocated space in living animals is not easy to determine, certain formulas have been developed to calculate the respective areas by the weight of the animal. Proposed by Petherick (7) and supported in additional studies (25, 37), all equations used transform bodyweight into space with the help of a space allowance coefficient. As this coefficient differs even for same body weight classes in these studies, likely based on variations in the study designs, different recommendations for space allowance were given if allometric formulas had been used (34, 37). In our study, a comparison of measured areas for the defined body positions with the results of three different formulas for three respective body positions described by Petherick and Phillips (25) was realized. The results clearly indicate that, especially with regards to “fully recumbent lying,” the equitation was not precise; even though both in measurements and in calculations, the fully recumbent body position requires the largest space, the equitation resulted in a difference of 0.544 m^2^ above the measured value of 0.486 ± 0.040 m^2^. These results clearly show that there is room for improvement in allometric formulas with respect to accuracy for finishing pigs in different body positions.

Regarding the comparison with recommended legal space requirements on transport vehicles in Europe, the average mean weight of the finishing pigs in this study was 109.01 ± 11.45 kg, about 10 kg higher than the weight mentioned in Council Regulation (EC) No 1/2005 (19), which is “around 100 kg.” The recommendation of a loading density of 235 kg/m^2^ was adapted to the actual mean weights of the different body position groups. In this context, it has to be emphasized that the considerations in this study are on a theoretical base. Under practical conditions, there is no individual calculation of the space offered per pig, rather according to the average weight of the loaded lot, usually estimated by the transportation staff.

For the best possible animal welfare on transport vehicles, not only the static space has to be taken into account, but also the additional remaining free space. When comparing the measured values with the legally required ones, it is important to keep in mind that only static space is considered by planimetric measurements. The deviations between measured and legally required space assessed in this study reflect the free space that is available for finishing pigs' dynamic and social space needs. However, knowledge or recommendations on these dynamic and social space needs, especially on transportation vehicles, are lacking. Particularly on long-distance transports, all pigs in the transportation vehicle should be able to lie down and get up with normal movements. With too little floor space available, the pigs are forced to stand very close to each other and to balance vehicle movements. Lying down in exhaustion can be impaired and the animals could fall over one another. These situations may increase stress, the number of injuries or even animal mortality (38–40). Moreover, animal body temperature can rise, resulting in discomfort or even in transport deaths (38). Crowding can also cause social pressure, resulting in conflicts and fights, or a constant change of body positions, which may lead to physiological reactions and exhaustion (22, 41). Therefore, sufficient space has to be provided to allow the animals to lie down (35) and evade other individuals. On the contrary, the provision of too much space may also be a risk factor, and hence should probably be avoided as well (26, 35). At low loading densities, the animals might not be able to keep the balance on turbulent route sections or during sudden braking and can be thrown through the vehicle (42).

Even though deviations for standing positions seem to hint on an adequate space provision, we cannot answer the question of whether the remaining free floor space is enough for an individual standing pig. For the space allocated for lying positions, the conclusion is clearer; deviations are much lower and even negative in the case of body position LL for the pigs examined in this study. Therefore, for lying finishing pigs, the minimal floor area offered on animal transportation vehicles according to European legislation is insufficient for modern finishing pigs.

## Conclusion

The results of this study reveal that finishing pigs of modern genetics are heavier than the average pig of 30 years ago, when scientific results on body dimensions first entered into legislation. It can be assumed also that the static space that is taken by the pigs has increased, as the weights are significantly related to this space. It was found that allometric estimations scientifically described for calculating the floor area physically covered by a pig's body are not consistently accurate. Especially for lying positions, allometric formulas need to be modified. Moreover, this data provides a basis for reflecting and discussing legal requirements for pig transport. It was shown that the minimal floor area offered on animal transportation vehicles, according to European legislation, is not sufficient to grant finishing pigs of modern genetic origin enough static space in the fully recumbent body position. Therefore, further data of body dimensions of single pigs, and especially groups of animals, are needed to determine the static space needs. Even more important, further studies on the behavior of pigs in transport situations have to be considered to increase our understanding of the required free space.

## Data Availability Statement

The datasets for this study are available on request. The raw data supporting the conclusions of this manuscript will be made available by the authors (without undue reservation) to any qualified researcher.

## Author Contributions

JH, BS, HA, and NK contributed to the study design. HA organized and conducted the field work. HA and NV performed the statistical analysis. BS supported the field work. HA prepared the original draft of the manuscript. NK reviewed and edited the first version of the manuscript. JH and NK supervised the project. JH and HA acquired the funding. All authors contributed to manuscript revisions, read, and approved the submitted version.

### Conflict of Interest Statement

The authors declare that the research was conducted in the absence of any commercial or financial relationships that could be construed as a potential conflict of interest.

## Supplementary Material

Supplementary Figure 1 Picture of the experimental set up on farm (Photo: H. Arndt).
