# Supplementary figures and images for: Do Pigs Have Adequate Space in Animal Transportation Vehicles?—Planimetric Measurement of the Floor Area Covered by Finishing Pigs in Various Body Positions
Source: Front Vet Sci. 2019 Jan 10;5:330. doi: 10.3389/fvets.2018.00330 (PMC6335254; doi:10.3389/fvets.2018.00330)

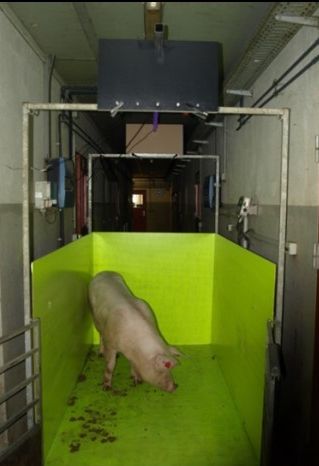

Supplement: Supplementary Figure 1 — Picture of the experimental set up on farm (Photo: H. Arndt). [file Image_1.JPEG]
